# Machine Learning for Predicting Chronic Renal Disease Progression in COVID-19 Patients with Acute Renal Injury: A Feasibility Study

**DOI:** 10.3390/biomedicines12071511

**Published:** 2024-07-08

**Authors:** Carlos Gracida-Osorno, Gloria María Molina-Salinas, Roxana Góngora-Hernández, Carlos Brito-Loeza, Andrés Humberto Uc-Cachón, José Ramón Paniagua-Sierra

**Affiliations:** 1 Servicio de Medicina Interna, Hospital General Regional No. 1, CMN Ignacio García Téllez, Instituto Mexicano del Seguro Social, Mérida 97150, Mexico; 2 Unidad de Investigación Médica Yucatán, Hospital de Especialidades, CMN Ignacio García Téllez, Instituto Mexicano del Seguro Social, Mérida 97150, Mexico; gmolina70@gmail.com (G.M.M.-S.); andresuccachon@gmail.com (A.H.U.-C.); 3 Facultad de Matemáticas, Universidad Autónoma de Yucatán, Mérida 97119, Mexico; tary83@gmail.com (R.G.-H.); carlos.brito@correo.uady.mx (C.B.-L.); 4 Unidad de Investigación Médica en Enfermedades Nefrológicas, Hospital de Especialidades, CMN Siglo XXI, Instituto Mexicano del Seguro Social, México City 06720, Mexico; jrpaniaguas@gmail.com

**Keywords:** acute kidney injury, boosting, chronic kidney disease progression, COVID-19, logistic regression, machine learning, prediction model, random forest, support vector machine

## Abstract

This study aimed to determine the feasibility of applying machine-learning methods to assess the progression of chronic kidney disease (CKD) in patients with coronavirus disease (COVID-19) and acute renal injury (AKI). The study was conducted on patients aged 18 years or older who were diagnosed with COVID-19 and AKI between April 2020 and March 2021, and admitted to a second-level hospital in Mérida, Yucatán, México. Of the admitted patients, 47.92% died and 52.06% were discharged. Among the discharged patients, 176 developed AKI during hospitalization, and 131 agreed to participate in the study. The study’s results indicated that the area under the receiver operating characteristic curve (AUC-ROC) for the four models was 0.826 for the support vector machine (SVM), 0.828 for the random forest, 0.840 for the logistic regression, and 0.841 for the boosting model. Variable selection methods were utilized to enhance the performance of the classifier, with the SVM model demonstrating the best overall performance, achieving a classification rate of 99.8% ± 0.1 in the training set and 98.43% ± 1.79 in the validation set in AUC-ROC values. These findings have the potential to aid in the early detection and management of CKD, a complication of AKI resulting from COVID-19. Further research is required to confirm these results.

## 1. Introduction

The incidence of acute kidney injury (AKI) triggered by COVID-19 varies from 0.5% to 58.6% depending on the different definitions employed and the various populations studied [1]. AKI in COVID-19 patients has multiple pathophysiological mechanisms, including direct tubular damage induced by the virus, loss of the brush border, toxic-induced tubular damage (rhabdomyolysis), non-ischemic vacuolar degeneration, glomerular damage, AKI associated with sepsis, and microvascular dysfunction (thrombotic disease with a high risk of fibrin thrombus formation in the pulmonary blood vessels and renal microvasculature) [2]. AKI increases the risk of developing chronic kidney disease (CKD) by eight times [3]. Therefore, it is crucial to identify patients at risk of CKD progression after AKI. Efforts have been made to use multivariate analysis to identify patients who will develop CKD; however, its usefulness is limited [4,5]. Modern statistical tools have been employed to identify cohorts of individuals within a population, encompassing artificial intelligence [6]. Given that the response variable was binary (CKD vs. no CKD), it was appropriate to utilize supervised learning classification, which is a type of artificial intelligence [7]. In this study, we investigated the feasibility of four supervised learning methods, namely support vector machine (SVM), random forest (RF), logistic regression (LR), and boosting, for the classification of patients who will progress to post-AKI CKD due to COVID-19.

## 2. Materials and Methods

### 2.1. Study Type and Design 

This study is a cross-sectional ambispective (merge historical data with future follow-ups, allowing researchers to analyze both pre-existing and newly collected information) [8], and a prolective approach (captures data in real-time during the occurrence of causal phenomenon; the baseline state is initially documented, then the intervention, and finally the outcome) [9]. 

### 2.2. Population, Setting, and Period of Study

The study population comprised all adult patients who were discharged to the community after being hospitalized at the Regional General Hospital of the National Medical Center of Ignacio García Téllez of Instituto Mexicano del Seguro Social (IMSS), a second-level hospital located in Mérida, Yucatán, México, for COVID-19 and AKI between 1 April 2020 and 31 March 2021.

The inclusion criteria for the study were as follows: patients aged ≥ 18 years who had been hospitalized for COVID-19, diagnosed by molecular or antigenic testing for SARS-CoV-2, developed AKI during their hospital stay, and agreed to participate by signing an informed consent form. The following were the exclusion criteria: patients who passed away during hospitalization or after being discharged from COVID-19, those who received renal replacement therapy before contracting COVID-19, those who underwent renal transplantation, those who experienced SARS-CoV-2 reinfection, those with incomplete data, those refused to participate, and those who withdrew from the study.

### 2.3. Definitions

The degree of severity of COVID-19 was defined according to the criteria established by the World Health Organization [10]:

- Asymptomatic was characterized by the absence of symptoms.
- Mild disease was defined as symptomatic patients who met the case definition for COVID-19, without evidence of viral pneumonia or hypoxia.
- Moderate disease involved clinical signs of pneumonia, including fever, cough, dyspnea, and fast breathing, but there were no signs of severe pneumonia, with an SpO2 level of 90% or higher while breathing room air.
- Severe disease involved clinical signs of pneumonia, along with one of the following: a respiratory rate of over 30 breaths per minute, severe respiratory distress, or an SpO2 level below 90% while breathing room air.
- Critical disease was characterized by the presence of acute respiratory distress syndrome (ARDS).

The estimated glomerular filtration rate (eGFR) was calculated using a blood sample obtained after at least 8 h of fasting to measure the serum creatinine levels. The eGFR was estimated using the equation formulated by the Chronic Kidney Disease Epidemiology Collaboration (CKD-EPI 2021) [11]. AKI was defined as follows.

- Stage 1: creatinine increase ≥ 0.3 mg/dL or ≥150–200% of the baseline.
- Stage 2: creatinine increase ≥ 200–300% of the baseline.
- Stage 3: creatinine increases of ≥300% of the baseline or a serum creatinine level > 4 mg/dL with a sudden increase of at least 0.5 mg/dL [12].

Progression to CKD was defined based on the criteria of the Kidney Disease Quality Outcomes Initiative (K-DOQI) as a decrease in the glomerular filtration rate, as measured by the CKD-EPI 2021 formula, of greater than 25 mL/min/1.73 m^2^ in two measurements with an interval of >3 months, accompanied by a negative change in CKD staging. CKD staging was determined based on the K-DOQI [13].

### 2.4. Data Source of AKI and CKD Progression

The data used in this study were extracted from the Epidemiological Capture System, which is a comprehensive hospital database. Retrospectively, clinical data were extracted and analyzed from the electronic health records of patients who were hospitalized with COVID-19. These records contain extensive information about patients, including their demographic characteristics, vital sign monitoring, laboratory tests, imaging tests, drug treatments, length of stay, and discharge or death records.

To estimate the renal function of patients before hospitalization, serum creatinine values were obtained from the Comprehensive Laboratory System between 7 and 365 days before hospitalization. Additionally, patients’ laboratory information was linked to their specific hospital stay to assess the reliability of the subject selection process and data collection. There are no therapeutic guidelines for the management of AKI in patients with COVID-19, and conventional care for this condition was employed. This involves maintaining an adequate hemodynamic status, avoiding the use of potentially nephrotoxic drugs, and closely monitoring both clinical and biochemical parameters. Extracorporeal renal therapy was not administered in any patient. To validate the accuracy of the data, a team of external personnel randomly selected 20% of the patient records by using a random table and confirmed the information.

This study enrolled patients who experienced AKI and were subsequently discharged from the hospital. These individuals were invited to participate in the study and were contacted by the study team via phone to discuss the purpose of the research and address any questions they may have had. On the day of their initial consultation, participants received a letter of informed consent. CKD progression was determined through two medical consultations that were held three months apart and included comprehensive clinical assessments, such as vital signs, anthropometric measurements, and physical examinations. Blood and urine samples were collected and analyzed, and the resulting data were documented in a data-collection format. Patients with CKD progression were identified by comparing the creatinine values obtained from discharge and prospective consultations. 

The follow-up period was structured as follows: two medical visits with a nephrologist were arranged for medical evaluation and data verification, with the first scheduled one month after hospital discharge and the second three months after the initial visit. At the initial consultation, the participants were provided with an informed consent letter and telephone number for contact in case of clinical discomfort or the need for external medical intervention. Throughout the follow-up period, patients followed the protocols established by the Secretary of Health of Mexico for the medical care of COVID-19 patients during a health contingency. If they missed any scheduled appointments, they were proactively located and followed up. 

### 2.5. Prediction Variables

The following predictor variables were obtained during hospitalization: (1) demographic features, including age, sex, and body mass index; (2) comorbidities, such as hypertension, diabetes, obesity, cardiovascular disease, and kidney disease; (3) information on the number of days in hospital, severity of COVID-19, and severity of AKI; (4) laboratory parameters, including hematological parameters, glucose, creatinine, blood urea nitrogen, sodium, potassium, total bilirubin, albumin, alanine transaminase, aspartate transaminase, and lactate dehydrogenase; and (5) therapeutic and clinical management, including mechanical ventilation and vasopressor use. It is important to note that all patients received the same medical and pharmacological care and treatment was not considered a variable.

### 2.6. Data Preprocessing

To mitigate the potential bias caused by missing data, variables with more than 20% missing values were not considered in the analysis. Of the 44 variables included, only 4 had missing values, accounting for more than 6%. We used the available values of the variable containing missing data to determine the median, which represented the central value in the dataset. Subsequently, we substituted the missing data with computed median values. Finally, the Z-score method was used to standardize the variables.

To ensure balanced data distribution among the groups, the random oversampling (ROS) and Tomek’s link subsampling methods were employed. ROS increases the minority class without losing information, whereas the Tomek’s link method identifies the majority class elements closest to the minority class and removes them to highlight class distinctions [14]. Feature selection is an integral part of model construction, and we implemented five variable selection methods to identify the most crucial features: ROC and *p*-value plots, SHAP (Shapley additive explanations, determine the significance of the features and their impact on the final predictions [15]), principal component analysis (uses uncorrelated variables called principal components to describe a dataset [16]), and forward and backward regression. Two additional regression-based selections were later developed and employed only the hospital admission data.

### 2.7. Data Analysis

Descriptive statistics were used to analyze the demographic and clinical features of the patients. Descriptive statistics were used to summarize the continuous variables (means and standard deviations, medians, interquartile ranges, or frequency counts and percentages).

Continuous variables were compared between patients with and without composite events using an unpaired Student’s *t*-test or Mann–Whitney U test, as appropriate, and categorical variables were compared using the chi-square test or Fisher’s exact test.

For supervised learning, four machine-learning algorithms (SVM, RF, LR, and boosting) were used to train the models. The data employed for the model development were randomly divided into two parts, with 80% of the data utilized for training and the remaining 20% for internal validation. The optimal parameters for each algorithm were selected by grid search using 10-fold cross-validation based on the performance metrics (with preference given to the highest possible area under the curve [AUC]).

SVM is a classification algorithm that employs a hyperplane to separate data into distinct classes with the objective of maximizing the margin between them. To achieve this, the algorithm identifies the hyperplane that provides the greatest margin by evaluating the distance between each observation and prospective hyperplane. Once the hyperplane with the maximum margin has been determined, the test observations can be classified by reference to the side of the hyperplane on which they are situated. SVM may also employ different kernels to address nonlinearity [17]. 

RF is a machine-learning algorithm utilized for classification that employs a strategy of constructing multiple decision trees on randomly selected subsets of the training data and then combining their predictions to formulate a final prediction. Each tree in the forest was established using a distinct subset of features and a different subset of training data to mitigate overfitting and enhance the accuracy of the model. The ultimate prediction was derived by tabulating the majority vote for all the trees in the forest [18].

RL is a statistical approach used for binary classification problems, in which the output variable can only attain two possible values. It assesses the probability of an event transpiring based on input variables using a sigmoid function. The parameters of the logistic function are determined via maximum likelihood estimation, which is a statistical method for determining the values of the parameters that maximize the likelihood of observing data [19]. 

Boosting is a machine-learning technique that merges multiple elementary models, known as weak learners, to create a more precise and intricate model. The procedure involves sequentially adding new weak learners to the model, with each new learner concentrating on the inaccuracies of the previous learners. This methodology enables the model to enhance its accuracy gradually over time [20].

### 2.8. Metrics for Evaluating Model Performance

To evaluate the effectiveness of the models, we calculated their sensitivity and specificity and used the AUC as a metric to compare the performance of the machine-learning algorithm models. Additionally, we employed secondary metrics for the machine-learning models, such as accuracy, precision, recall, and F1-score, to further evaluate their performance. Accuracy measures the proportion of correctly predicted outcomes, precision represents the proportion of correctly identified positive cases among all the predicted positives, the recall score assesses the model’s ability to identify positive instances from actual positives, and the F1-score, which is the harmonic mean of precision and sensitivity, provides a comprehensive measure of the model’s overall output quality [21]. 

Analyses were conducted utilizing IBM SPSS Statistics version 24 and Python 3.11.3, which were obtained from the Python Software Foundation (Python 3.11.4 64-bit|Qt 5.15.2|PyQt5 5.15.7|Windows 10), available at http://www.python.org, accessed on 14 August 2023.

## 3. Results

### 3.1. Population, Setting, and Period of Study

**Figure 1:**
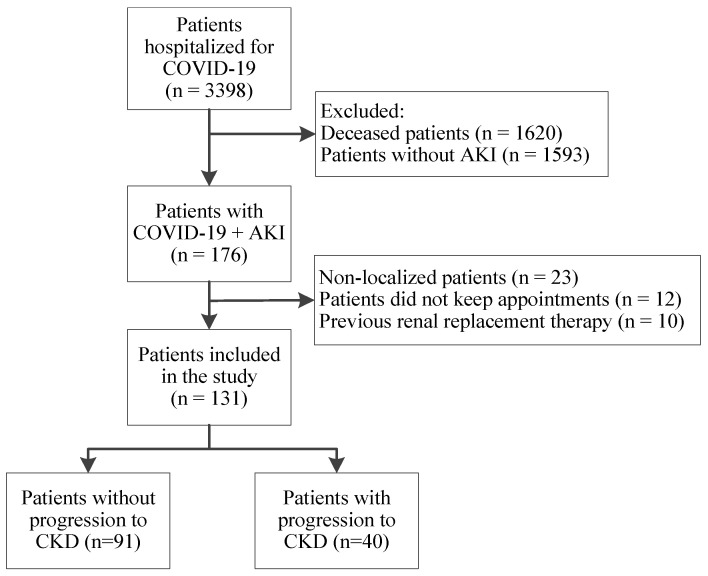
The flowchart of patient selection.

Between 1 April 2020 and 31 July 2021, 3398 patients were admitted to our hospital with COVID-19. Of these, 1620 (47.92%) unfortunately passed away, while 1769 (52.06%) were discharged. We thoroughly examined the medical records and laboratory results of the discharged patients to identify those who developed AKI during hospitalization for COVID-19. A total of 176 (176/1769; 9.9%) patients having AKI met the rest of the inclusion criteria, but just 131 (131/176; 74.4%) consented to participate in our study and completed the follow-up process (Figure 1). Of our study population, 40 patients progressed to CKD (40/131; 30.5%).

### 3.2. Clinical and Epidemiological Characteristics of the Cohort 

The relevant clinical, epidemiological, and laboratory findings are presented in Table 1 and Table S1. The study had an average patient age of approximately 61 years, with no major age discrepancies between the two groups (*p* = 0.167). The sex distribution was also comparable in both groups (*p* = 0.917), with approximately 62% of patients being male. Additionally, the non-progression group had a slightly higher body mass index (BMI), but this difference was not statistically significant (*p* = 0.162). The group that progressed to CKD had a higher prevalence of diabetes (57.5% vs. 33%, *p* = 0.008) and a lower prevalence of obesity (52.5% vs. 70.3%, *p* = 0.049). In addition, a history of kidney disease was more common in the group that progressed to CKD (50% vs. 27.5%, *p* = 0.012). There were no significant differences in the COVID-19 severity grades between the two groups (*p* = 0.343); however, kidney disease severity at Stage 3 was significantly different, with a higher percentage of patients in the progression group at this stage (20% vs. 5.5%, *p* = 0.033). There were no significant differences in the number of days spent on oxygen or in the hospital between the two groups. It is worth noting that patients who progressed to CKD had lower levels of hemoglobin during hospitalization (*p* = 0.008), and at discharge, their lymphocyte percentage decreased significantly (*p* = 0.003). Furthermore, at discharge, their glucose levels were elevated (*p* = 0.025) and creatinine levels were elevated (*p* = 0.006) compared with those in patients who did not progress to CKD.

### 3.3. Development and Internal Validation of the Predictive Models 

Tables S2, S3, and Table 2 present a detailed analysis of the training, testing, and validation of the performance metrics of the multiple algorithms measured using various subsets of variables. The first column indicates the algorithms and specific subset of variables utilized for each algorithm, whereas the following seven columns display the accuracy, precision, recall, F1-score, sensitivity, specificity, and AUC results for each algorithm.

The results of the classifier implemented using ROS are noteworthy. In general, the SVM and boosting models performed exceptionally well for all of the metrics and sets of variables. For example, the SVM model achieved an average AUC of 91.58%, whereas the RF model achieved an average AUC of 87.22%. The performance of the RL model was relatively low, with an average AUC of 76.98%. The performance of the boosting model was moderate, with certain metrics exhibiting high values and others exhibiting low values. The overall average AUC was 82.88%. The Tomek’s link subsampling method displayed good results, although its performance in each category was not as good as that of ROS. The results of the Tomek’s link method are not included in the analysis.

**Figure 2:**
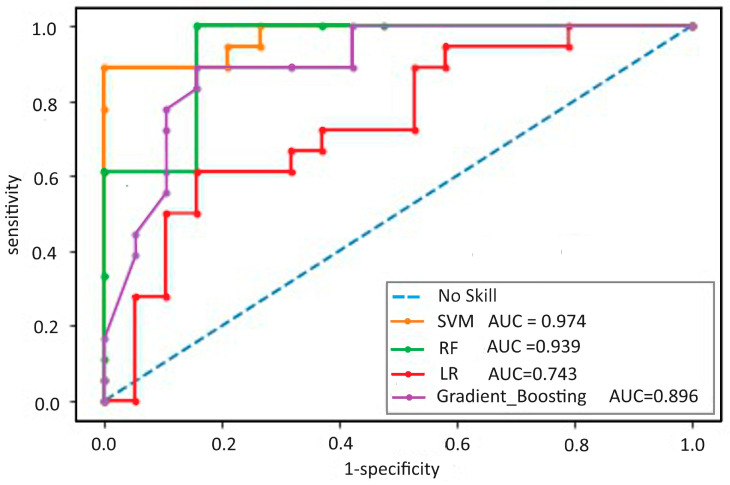
Receiver operating characteristic curve of the four models; method of variable selection ROC. SVM: support vector machine; RF: random forest; LR: logistic regression; AUC: area under the curve.

Regarding the variable selection methods, the models tended to perform better on the “ROC” variable selection set (Figure 2). The SVM model achieved in the training cohort an AUC of 99.88% (0.11) for predicting composite outcomes and calibration [Brier score: 0.11, Hosmer–Lemeshow test *p* value = 0.75]. Internal validation confirmed the good discriminability of the predictive model, with a similar AUC of 98.43% (1.79) for predicting composite outcomes and calibration [Brier score: 0.11, Hosmer–Lemeshow test *p* value = 0.63] (Table 3).

## 4. Discussion

In this study, a predictive model for CKD progression in patients with AKI resulting from COVID-19 was developed using machine learning. The sample comprised a higher proportion of men than women (61.8%), which is consistent with the findings of Silver et al. (58.2%) [22]. More than half of the patients had high blood pressure and almost two-thirds were obese, as reported by van Son et al. (71%) [23]. The severity of COVID-19 was highest at Level 4, accounting for more than half of the cases.

Severe AKI was present in approximately one-third of the patients analyzed, and a few patients experienced cardiovascular events during the disease. Notably, the selected patients were COVID-19 survivors with AKI; thus, it is possible that the incidence of cardiovascular conditions was higher in these patients.

Some conditions that can explain the progression to CKD in our patients are as follows: (a) A decrease in the number of nephrons results in an increased filtration rate of the remaining nephrons, leading to elevated filtration pressure. This phenomenon, known as remnant nephron hypertrophy, can cause increased shear stress in podocytes, potentially resulting in their detachment. When this detachment occurs, it can lead to focal and segmental glomerulosclerosis, which can ultimately result in global glomerulosclerosis and nephron atrophy [24,25]; (b) Anemia, which is characterized by a low concentration of hemoglobin in the circulation, results in a reduced oxygen-carrying capacity, leading to tissue hypoxia and potentially impairing renal oxygenation, and is considered a non-conventional factor in CKD progression [26,27,28]; (c) Patients with CKD typically exhibit reduced lymphocyte counts, a characteristic sign associated with increased inflammation. Moreover, a decline in renal function over time leads to the activation and selective loss of T cells and CD4+ cells, while CD8+ cells significantly increase [29,30]; (d) Glucose filtration and glomerular hyperfiltration lead to tubular hyperreabsorption of glucose and sodium, resulting in glomerular and tubular hypertrophy. Over time, this can result in glomerulosclerosis and tubule atrophy [31].

To ensure the highest accuracy of the classifier, we purposefully excluded variables with missing values that were originally intended to be included in the model. These variables, such as D-dimer, were not mandatory to study in all COVID-19 hospitalized patients, even though they have been linked to a poor prognosis [32]. 

To enhance the utility of the classifier in a clinical setting, it is recommended to restrict the number of variables incorporated into the classifier. In this study, we evaluated the stability of the models under various scenarios using seven variable-selection methods. The results revealed that ROC analysis was the most effective method for selecting the optimal variables. This may be attributed to the fact that the ROC curve is less prone to variations in the scale and threshold of probabilities, thereby making it a reliable method for variable selection [33].

Of the classifiers utilized, the SVM generated the most favorable results. One explanation for the superior performance of the SVM is its capacity to effectively manage the nonlinear relationships between the features and target variables. Decision trees, on the other hand, rely on linear relationships, and can therefore struggle with nonlinear relationships [34,35,36,37]. In addition, the SVM is more robust to outliers than LR, RF, and boosting. These methods are sensitive to data values [38]. SVM demonstrates advantages over other learning methods and is often preferable for patient classification tasks because of its ability to handle nonlinear relationships and its robustness to outliers.

When interpreting the findings of the study, it is of the utmost importance to consider the following factors: (1) urine output was not included in the definition of AKI; (2) the study population did not receive COVID-19 vaccination, which may have influenced the natural progression of the disease; (3) the results only pertained to patients who survived hospitalization for COVID-19 and not those who were admitted and subsequently passed away; and (4) while the models displayed exceptional performance in predicting the progression to CKD during training and internal validation, external validation is necessary to confirm their accuracy.

To date, most clinical studies that have employed machine-learning methodologies have focused on the development and validation of risk classification models for predicting the occurrence of AKI in hospitalized patients [39,40,41,42,43] and the onset of AKI in critically ill patients in intensive care units [44,45]. Additionally, some studies have addressed chronic outcomes such as death at discharge, initiation, or discontinuation of renal replacement therapy, or specific CKD stages [46,47,48,49,50]. However, studies on CKD progression at all stages after AKI, which is a major adverse kidney condition, have received limited attention. The classification methods that have been studied can be applied to various entities that contribute to a decline in kidney function, such as post-surgery kidney cancer, by incorporating predictor variables such as preoperative proteinuria [51], thereby expanding their range of application.

Post-hospitalization AKI patient follow-up represents a significant challenge owing to the combination of two factors: the process is both time-consuming and expensive, and patients tend to drop out of follow-up care [52]. It is crucial to address these challenges because CKD is a potentially dangerous condition that requires careful management. The utilization of these risk classification tools may be critical for targeting strategies to optimize the care of high-risk progressive CKD patients in specialized post-discharge clinics, which can aid in the promotion of kidney and critical illness recovery and enhance patient-centered outcomes that are more likely to benefit from the intervention [53,54]. Lastly, the clinical significance of the proposed method lies in its potential to accurately identify high-risk individuals for CKD using readily available information prior to hospital discharge. This enables health care providers to make informed decisions about future care. The development of a user-friendly application (app) for convenient access to information and its appropriate categorization is a future possibility. 

## 5. Conclusions

In this study, we showed that the four classifiers (SVM, RF, LR, and boosting) successfully identified patients who developed CKD after AKI caused by COVID-19. The SVM model demonstrated the best overall performance, achieving a classification rate of 99.8% ± 0.1 in the training set and 98.43% ± 1.79 in the validation set. These results were obtained using the ROS-balancing method and the selection of variables using the ROC. The models analyzed are promising for medical decision making; however, further research in this field is required.

## Figures and Tables

**Table 1:** Distribution of the clinical and laboratory variables ^a^ between the non-CKD progression and CKD progression.

Clinical and Laboratory Variables	Total, AKI (n = 131)	No Progression to CKD (n = 91)	Progression to CKD (n = 40)	*p*
Age (years), M (P_25_–P_75_)	61.0 (51.0–70.0)	59.0 (47.0–70.0)	63.0 (55.0–69.5)	0.167
Male, n (%)	81 (61.8)	56 (61.5)	25 (62.5)	0.917
BMI (kg/m^2^), M (P_25_–P_75_)	31.6 (27.4–35.1)	32.0 (27.6–35.3)	30.2 (26.6–35.0)	0.162
Comorbidities, n (%)				
Diabetes	53 (40.5)	30 (33.0)	23 (57.5)	0.008
Hypertension	74 (56.5)	49 (53.8)	25 (62.5)	0.358
Obesity	85 (64.9)	64 (70.3)	21 (52.5)	0.049
History of kidney disease	45 (34.4)	25 (27.5)	20 (50.0)	0.012
COVID-19 metrics				
COVID-19 severity, n (%)				0.343
Asymptomatic	10 (7.6)	7 (7.7)	3 (7.5)	
Mild disease	11 (8.4)	7 (7.7)	4 (10.0)	
Moderate disease	30 (22.9)	21 (23.3)	9 (22.5)	
Severe disease	71 (54.2)	47 (51.6)	24 (60.0)	
Critical disease	9 (6.9)	9 (9.9)	0	
Kidney disease severity (Stage 3), n (%)				0.033
1	82 (62.6)	61 (67.0)	21 (52.5)	
2	36 (27.5)	25 (27.5)	11 (27.5)	
3	13 (9.9)	5 (5.5)	8 (20.0)	
Cardiovascular event, n (%)	9 (6.9)	7 (7.7)	2 (5.0)	0.575
Number of days on oxygen, M (P_25_–P_75_)	9.0 (3.0–15.0)	9.0 (3.0–14.0)	10 (3–15)	0.672
Number of days in hospital, M (P_25_–P_75_)	15.48 (13.47–17.49)	13.0 (8.0–21.0)	13 (9–17)	0.785
Laboratory variables admission, M (P_25_–P_75_)				
Hemoglobin (g/dL)	13.2 (12.0–14.7)	13.8 (12.3–15.0)	12.7 (11.0–13.8)	0.008
Hematocrit (%)	40.0 (36.0–45.0)	41.0 (37.0–45.0)	39.0 (32.2–42.0)	0.008
Creatinine (mg/dL)	1.2 (0.9–1.8)	1.1 (0.9–1.5)	1.5 (1.1–2.1)	0.012
Clearance of creatinine (ml/min)	61.0 (36.0–82.5)	63.0 (39.0–85.8)	46.6 (29.0–68.9)	0.019
Laboratory variables discharge, M (P_25_–P_75_)				
Neutrophils (%)	74.0 (65.0–80.0)	72.0 (64.0–67.0)	78.0 (72.0–81.0)	0.012
Lymphocytes (%)	17.0 (12.0–24.0)	18.0 (13.0–25.0)	13.0 (11.0–19.2)	0.003
Glucose (mg/dL)	107.0 (89.0–137.0)	103 (87.0–123.0)	120.5 (94.0–183.0)	0.025
Creatinine (mg/dL)	0.9 (0.7–1.1)	0.8 (0.6–1.0)	1.0 (0.8–1.2)	0.006
Creatinine clearance (ml/min)	90.5 (65.8–101.3)	94.0 (74.0–104.0)	78 (55.9–97.4)	0.010

^a^ statistically significant.

**Table 2:** Concentration of validation results of classifiers with ROS rolling.

								Validation							
		Accuracy		Precision		Recall		F1-Score		Sensitivity		Specificity		AUC	
All variables (44)	SVM	81.62	1.71	83.56	4.78	80.53	4.33	81.81	1.51	80.53	4.33	82.78	6.11	90.15	2.70
	RF	79.46	3.42	76.69	3.03	86.32	5.66	81.14	3.49	86.32	5.66	72.22	4.54	88.06	1.53
	RL	68.65	4.80	66.98	3.73	76.84	8.67	71.41	5.21	76.84	8.67	60.00	6.31	66.58	3.79
	Boosting	72.70	5.62	69.61	5.93	84.74	6.77	76.17	4.23	84.74	6.77	60.00	11.94	77.65	4.73
ROC (8)	SVM	93.22	1.74	100.00	0.00	88.05	3.06	93.62	1.76	88.05	3.06	100.00	0.00	98.43	1.79
	RF	80.81	2.37	76.11	2.99	91.58	2.72	83.08	1.80	91.58	2.72	69.44	5.40	89.88	2.76
	RL	65.95	2.28	65.21	1.47	72.11	5.58	68.41	3.10	72.11	5.58	59.44	2.68	72.11	0.50
	Boosting	74.05	5.58	70.50	5.81	86.32	7.53	77.36	4.78	86.32	7.53	61.11	11.11	78.95	6.97
SHAP (11)	SVM	92.16	2.37	100.00	0.00	84.74	4.61	91.68	2.79	84.74	4.61	100.00	0.00	93.68	2.61
	RF	79.73	3.43	79.01	4.77	83.16	7.36	80.73	3.57	83.16	7.36	76.11	7.43	85.01	3.08
	RL	70.54	0.85	65.93	1.14	88.42	3.33	75.49	0.98	88.42	3.33	51.67	3.75	73.30	0.76
	Boosting	83.51	4.12	77.44	2.61	95.79	7.77	85.53	4.18	95.79	7.77	70.56	3.75	85.70	4.37
PCA (24)	SVM	85.14	1.42	100.00	0.00	71.05	2.77	83.05	1.90	71.05	2.77	100.00	0.00	89.04	2.42
	RF	77.84	4.19	78.36	3.12	78.42	7.21	78.29	4.87	78.42	7.21	77.22	3.15	86.54	3.75
	RL	72.97	2.55	70.16	2.59	82.63	2.54	75.86	2.07	82.63	2.54	62.78	4.57	77.78	1.51
	Boosting	77.57	5.26	75.20	6.43	85.26	5.44	79.69	4.12	85.26	5.44	69.44	10.88	80.45	5.28
LR forward (10)	SVM	92.43	2.79	100.00	0.00	85.26	5.44	91.96	3.27	85.26	5.44	100.00	0.00	93.99	2.02
	RF	78.11	3.70	75.00	4.99	86.84	2.77	80.36	2.69	86.84	2.77	68.89	8.36	86.14	3.03
	RL	74.59	1.89	72.69	2.08	81.05	2.72	76.61	1.71	81.05	2.72	67.78	3.51	79.62	1.61
	Boosting	71.89	3.65	67.91	3.62	86.32	5.08	75.92	3.05	86.32	5.08	56.67	6.83	82.95	3.43
LR backward (14)	SVM	90.54	1.42	100.00	0.00	81.58	2.77	89.83	1.68	81.58	2.77	100.00	0.00	90.86	1.76
	RF	77.03	2.92	75.28	2.85	82.63	7.46	78.58	3.46	82.63	7.46	71.11	5.74	85.64	3.30
	RL	67.30	4.50	68.07	4.25	68.42	5.55	68.20	4.58	68.42	5.55	66.11	4.86	76.78	1.28
	Boosting	81.08	4.41	75.85	4.06	93.16	8.25	83.41	4.22	93.16	8.25	68.33	7.88	85.77	4.79
LR forward admission (9)	SVM	80.54	3.99	81.52	4.18	80.53	6.10	80.89	4.18	80.53	6.10	80.56	5.40	86.41	2.88
	RF	74.59	3.17	74.06	3.24	77.89	4.15	75.88	3.06	77.89	4.15	71.11	4.38	85.26	2.35
	RL	72.97	3.37	70.31	2.96	82.11	5.08	75.69	3.29	82.11	5.08	63.33	4.68	77.69	2.40
	Boosting	74.59	5.58	71.14	5.84	86.32	9.35	77.66	5.15	86.32	9.35	62.22	11.65	78.82	6.01
LR backward admission (12)	SVM	87.03	3.07	100.00	0.00	74.74	5.98	85.42	4.04	74.74	5.98	100.00	0.00	89.02	3.85
	RF	73.51	5.81	74.59	7.17	74.74	10.76	74.15	6.12	74.74	10.76	72.22	10.48	82.06	4.44
	RL	66.76	3.13	67.74	2.78	67.37	5.98	67.46	3.71	67.37	5.98	66.11	4.10	78.25	0.74
	Boosting	74.05	5.28	68.46	5.27	93.16	7.46	78.67	4.04	93.16	7.46	53.89	10.81	83.17	4.08

Average performance metrics in % (accuracy, precision, etc.) for binary classification to predict CKD progression in patients with AKI secondary to COVID-19. SVM: support vector machine; RF: random forest; LR: logistic regression; ROC: receiver operating characteristic; SHAP: Shapley additive explanations; PCA: principal component analysis; AUC: area under the curve.

**Table 3:** Performance of the novel predictive model for composite outcome: method SVM and ROC variable selection.

	Discrimination Performance	Calibration Performance		Sensitivity	Specificity
Dataset	AUC (95% CI)	Brier Score	Hosmer–Lemeshow Test		
Training cohort	99.88 (0.11)	0.011	0.751	97.66 (1.81)	99.93 (0.46)
Validation cohort	98.43 (1.79)	0.011	0.635	88.05 (3.06)	100 (0)

AUC: area under the precision-recall curve; CI: confidence interval.

## Data Availability

The datasets generated and/or analyzed during the current study are not publicly available because they are the property of the Instituto Mexicano del Seguro Social. Institutional and federal dispositions restrict unlimited access to personal data, but they are available from the corresponding authors upon reasonable request with prior authorization from the institution.

## Supplementary Materials

The following supporting information can be downloaded at: https://www.mdpi.com/article/10.3390/biomedicines12071511/s1, Table S1: Distribution of the laboratory variables between the no CKD progression and CKD progression; Table S2: Concentration of training results of classifiers with ROS rolling; Table S3: Concentration of test results for classifiers with ROS rolling.
