# Supplementary material for: Machine Learning for Predicting Chronic Renal Disease Progression in COVID-19 Patients with Acute Renal Injury: A Feasibility Study
Source: Biomedicines. 2024 Jul 8;12(7):1511. doi: 10.3390/biomedicines12071511 (PMC11274434; doi:10.3390/biomedicines12071511)
Supplement: Supplementary file 1 [file biomedicines-12-01511-s001.zip › biomedicines-3003202-supplementary.pdf]

Table S1. Distribution of the laboratory variables between the non-CKD progression and CKD progression.

Clinical and laboratory variables	Total, AKI (n = 131)	No progression to CKD (n = 91)	Progression to CKD (n = 40)	*p*
Laboratory variables admission, M (P_{25}-P_{75})				
Hemoglobin (g/dL)	13.2 (12.0-14.7)	13.8 (12.3-15.0)	12.7 (11.0-13.8)	0.008
Hematocrit (%)	40.0 (36.0-45.0)	41.0 (37.0-45.0)	39.0 (32.2-42.0)	0.008
VCM (fL)	89.0 (86.0-92.0)	89.0 (86.0-91.0)	89.0 (84.2-92.0)	0.686
HCMC (pg)	32.0 (31.0-33.0)	32.0 (31.0-33.0)	32.0 (32.0-33.0)	0.321
Platelets (10^3AI)	306.0 (223.0-366.0)	306 (222-360)	306.0 (224.5-392.5)	0.582
Leukocytes (10^3AI)	10100 (7770-14000)	10230 (7830-14050)	9790 (7672-13490)	0.924
Neutrophil (%)	83.0 (78.0-89.0)	83.0 (78.0-89.0)	83.5 (76.7-89.7)	0.476
Lymphocytes (%)	10.0 (6.0-14.0)	10.0 (6.0-14.0)	10.0 (6.0-13.0)	0.590
Glucose (mg/dL)	128.0 (106.0-220.0)	125.0 (105.0-211.0)	166.0 (110.5-271.0)	0.125
Urea (mg/dL)	53.0 (41.0-79.0)	53.0 (39.0-75.0)	60.0 (43.0-102.0)	0.052
Creatinine (mg/dL)	1.2 (0.9-1.8)	1.1 (0.9-1.5)	1.5 (1.1-2.1)	0.012
Clearance of creatinine (ml/min)	61.0 (36.0-82.5)	63.0 (39.0-85.8)	46.6 (29.0-68.9)	0.019
Sodium (mmol/L)	138 (135-140)	138 (135-141)	138 (134-140)	0.521
Potassium (mmol/L)	4.5 (4.1-5.0)	4.5 (4.1-4.9)	4.5 (4.2-4.5)	0.711
ALT (U/L)	43.0 (29.0-67.0)	43.0 (31.0-70.0)	39.5 (28.0-59.7)	0.199
AST (U/L)	49.0 (39.0-47.0)	49.0 (40.0-78.0)	49 (36-74)	0.261
Total bilirubin's (U/L)	0.7 (0.6-0.9)	0.7 (0.6-0.9)	0.7 (0.7-0.9)	0.412
DHL (U/L)	464.0 (374.0-563.0)	464.0 (378.0-584.0)	464.0 (329.7-506.2)	0.328
Albumin (g/dL)	3.5 (3.2-3.7)	3.5 (3.3-3.8)	3.5 (3.1-3.7)	0.101
Laboratory variables discharge, M (P_{25}-P_{75})				
Hemoglobin (g/dL)	13.3 (12.0-14.3)	13.3 (12.2-14.4)	13.1 (10.6-14.05)	0.180
Hematocrit (%)	40.0 (37.0-44.0)	40.0 (38.0-44.0)	40.5 (32.0-43.0)	0.235
VCM (fL)	89.0 (86.0-92.0)	89.0 (86.0-92.0)	89.5 (86.0-92.0)	0.823
HCMC (pg)	32.0 (31.0-33.0)	32.0 (31.0-33.0)	32.0 (31.0-33.0)	0.907
Platelets (10^3AI)	333.0 (259.0-452.0)	333.0 (259.0-448.0)	314.0 (258.2-477.2)	0.907
Leukocytes (10^3AI)	9970 (7440-11800)	9680 (7300-11580)	10230 (7675-12842)	0.189
Neutrophils (%)	74.0 (65.0-80.0)	72.0 (64.0-67.0)	78.0 (72.0-81.0)	0.012
Lymphocytes (%)	17.0 (12.0-24.0)	18.0 (13.0-25.0)	13.0 (11.0-19.2)	0.003
Glucose (mg/dL)	107.0 (89.0-137.0)	103 (87.0-123.0)	120.5 (94.0-183.0)	0.025
Creatinine (mg/dL)	0.9 (0.7-1.1)	0.8 (0.6-1.0)	1.0 (0.8-1.2)	0.006
Creatinine clearance (ml/min)	90.5 (65.8-101.3)	94.0 (74.0-104.0)	78 (55.9-97.4)	0.010
Sodium (mmol/L)	137 (136-140)	137 (136-140)	137 (135-138)	0.744
Potassium (mmol/L)	4.5 (4.1-5.1)	4.4 (4.1-4.9)	4.7 (4.1-5.2)	0.237

Table S2. Concentration of training results of classifiers with ROS rolling.

		Train													
		**Acurracy**		**Precision**		**Recall**		**F1-Score**		**Sensitivity**		**Specificity**		**AUC**	
All variables (44)	SVM	98.21	1.66	99.05	1.42	97.36	3.67	98.15	1.77	97.36	3.67	99.04	1.45	99.89	0.13
	RF	98.21	1.31	97.82	1.92	98.61	0.93	98.21	1.30	98.61	0.93	97.81	1.96	99.83	0.17
	RL	85.45	2.09	83.72	3.57	88.06	3.15	85.75	1.90	88.06	3.15	82.88	4.71	90.36	1.54
	Boosting	97.38	0.96	96.49	1.57	98.33	1.71	97.39	0.96	98.33	1.71	96.44	1.61	98.46	0.95
ROC (8)	SVM	98.78	0.87	99.81	0.50	97.66	1.81	98.71	0.93	97.66	1.81	99.83	0.46	99.88	0.11
	RF	98.34	0.67	97.69	1.42	99.03	0.94	98.35	0.66	99.03	0.94	97.67	1.45	99.64	0.31
	RL	66.28	1.05	67.27	2.02	62.78	2.60	64.88	1.08	62.78	2.60	69.73	3.62	74.00	0.40
	Boosting	97.66	1.90	97.14	2.32	98.19	1.61	97.66	1.89	98.19	1.61	97.12	2.37	98.57	1.64
SHAP (11)	SVM	98.83	0.73	100.00	0.00	97.64	1.47	98.80	0.76	97.64	1.47	100.00	0.00	99.90	0.07
	RF	98.00	1.43	98.35	1.66	97.64	2.54	97.97	1.47	97.64	2.54	98.36	1.68	99.39	0.71
	RL	69.72	1.61	70.48	2.50	67.36	1.35	68.85	1.22	67.36	1.35	72.05	3.49	78.05	0.43
	Boosting	97.45	1.17	96.80	2.51	98.19	1.86	97.46	1.16	98.19	1.86	96.71	2.60	98.95	0.71
PCA (24)	SVM	98.90	0.74	100.00	0.00	97.78	1.49	98.87	0.76	97.78	1.49	100.00	0.00	99.92	0.06
	RF	98.00	0.60	97.97	1.79	98.06	1.49	97.99	0.60	98.06	1.49	97.95	1.85	99.78	0.25
	RL	68.14	1.71	68.63	3.28	66.67	3.27	67.51	1.11	66.67	3.27	69.59	5.88	75.23	0.40
	Boosting	98.21	0.87	97.16	1.31	99.31	0.73	98.22	0.86	99.31	0.73	97.12	1.36	98.78	0.90
LR forward (10)	SVM	98.48	1.48	100.00	0.00	96.94	2.99	98.43	1.59	96.94	2.99	100.00	0.00	99.91	0.06
	RF	96.41	1.77	96.16	2.21	96.67	1.76	96.40	1.78	96.67	1.76	96.16	2.22	99.16	0.63
	RL	77.59	1.31	75.32	1.30	81.67	3.63	78.32	1.63	81.67	3.63	73.56	2.51	84.41	0.65
	Boosting	97.31	1.47	96.36	2.06	98.33	1.94	97.32	1.47	98.33	1.94	96.30	2.15	98.38	1.37
LR backward (14)	SVM	99.45	0.44	100.00	0.00	98.89	0.88	99.44	0.44	98.89	0.88	100.00	0.00	99.96	0.04
	RF	97.86	0.83	97.95	1.68	97.78	1.76	97.85	0.84	97.78	1.76	97.95	1.74	99.66	0.37
	RL	74.21	1.39	74.27	2.19	73.75	3.73	73.93	1.66	73.75	3.73	74.66	3.61	82.67	0.62
	Boosting	96.48	0.95	95.45	2.10	97.64	1.86	96.50	0.92	97.64	1.86	95.34	2.26	97.69	1.20
LR forward admission (9)	SVM	96.41	1.33	97.33	1.19	95.42	2.78	96.34	1.41	95.42	2.78	97.40	1.20	99.51	0.30
	RF	97.79	1.12	97.68	1.68	97.92	1.88	97.78	1.14	97.92	1.88	97.67	1.71	99.52	0.36
	RL	77.17	1.82	74.80	2.26	81.67	4.33	78.00	2.00	81.67	4.33	72.74	3.90	82.75	0.41
	Boosting	96.62	1.76	95.24	2.90	98.19	1.61	96.67	1.68	98.19	1.61	95.07	3.18	98.33	1.08
LR backward admission (12)	SVM	99.03	0.74	100.00	0.00	98.06	1.49	99.01	0.76	98.06	1.49	100.00	0.00	99.94	0.09
	RF	98.00	1.28	97.56	1.97	98.47	1.22	98.00	1.26	98.47	1.22	97.53	2.02	99.62	0.49
	RL	70.83	1.38	71.81	1.75	68.06	3.93	69.81	1.98	68.06	3.93	73.56	3.03	78.54	0.58
	Boosting	94.62	1.52	93.98	2.80	95.42	3.53	94.62	1.56	95.42	3.53	93.84	3.05	97.84	1.22

Average performance metrics in % (accuracy, precision, etc.) for binary classification to predict CKD progression in patients with AKI secondary to COVID-19. SVM: support vector machine; RF: random forest; LR: logistic regression; ROC: receiver operating characteristic; SHAP: shapley additive explanations; PCA: principal component analysis; AUC: area under the curve.

Table S3. Concentration of test results for classifiers with ROS rolling.

		Test													
		**Acurracy**		**Precision**		**Recall**		**F1-Score**		**Sensitivity**		**Specificity**		**AUC**	
All variables (44)	SVM	81.90	17.25	91.24	12.24	74.37	33.81	75.57	28.33	74.37	33.81	90.97	13.69	88.56	14.66
	RF	82.38	12.50	79.67	16.92	84.78	11.30	81.75	13.60	84.78	11.30	79.86	16.32	89.06	8.11
	RL	66.38	10.62	68.54	19.30	75.26	20.42	67.90	11.34	75.26	20.42	60.62	26.36	67.48	14.03
	Boosting	73.86	9.54	71.05	12.54	83.31	16.82	75.22	8.89	83.31	16.82	63.64	17.20	82.13	8.43
ROC (8)	SVM	87.82	8.64	97.68	6.22	77.45	16.23	85.32	10.66	77.45	16.23	98.43	4.25	87.22	11.08
	RF	83.57	6.43	80.06	11.74	89.78	9.92	83.83	6.68	89.78	9.92	77.22	14.17	91.10	5.85
	RL	64.19	11.05	66.77	21.07	59.62	21.00	60.58	14.98	59.62	21.00	70.79	19.45	72.46	18.99
	Boosting	78.19	16.24	74.40	19.04	83.12	16.86	78.00	17.24	83.12	16.86	73.24	18.24	83.10	16.56
SHAP (11)	SVM	88.24	7.16	100.00	0.00	77.38	11.97	86.78	7.72	77.38	11.97	100.00	0.00	89.78	6.76
	RF	82.76	11.96	84.31	14.10	83.08	19.97	81.97	12.83	83.08	19.97	84.54	15.61	89.93	10.49
	RL	67.81	10.93	70.77	22.34	65.77	12.52	66.30	12.61	65.77	12.52	70.83	23.32	76.34	11.64
	Boosting	77.95	9.98	76.51	18.56	84.78	13.16	78.72	10.51	84.78	13.16	73.33	20.25	84.49	10.65
PCA (24)	SVM	88.86	7.67	100.00	0.00	78.12	14.11	87.10	8.61	78.12	14.11	100.00	0.00	91.08	6.69
	RF	81.38	5.65	81.89	14.31	83.95	12.46	81.36	5.89	83.95	12.46	81.11	16.15	91.03	6.52
	RL	64.33	11.72	67.68	20.95	65.97	18.72	63.83	12.01	65.97	18.72	66.49	25.76	72.13	10.82
	Boosting	82.14	8.47	76.37	9.96	93.25	7.29	83.72	7.85	93.25	7.29	70.78	13.70	86.08	10.01
LR forward (10)	SVM	85.33	13.29	100.00	0.00	72.38	20.24	82.18	17.19	72.38	20.24	100.00	0.00	90.33	6.56
	RF	74.57	12.49	71.47	16.36	78.67	16.45	74.29	14.64	78.67	16.45	71.63	13.60	83.87	12.18
	RL	71.86	9.61	72.86	17.48	76.09	20.81	71.41	12.43	76.09	20.81	69.17	24.40	78.73	14.59
	Boosting	73.38	14.30	69.33	15.17	83.69	18.56	74.70	14.31	83.69	18.56	64.64	19.57	78.63	13.28
LR backward (14)	SVM	94.48	4.29	100.0	0.00	89.40	7.94	94.24	4.38	89.40	7.94	100.0	0.00	95.95	4.15
	RF	79.29	8.10	80.31	13.67	79.94	17.16	78.36	10.56	79.94	17.16	81.49	13.72	91.11	6.38
	RL	65.76	13.54	72.83	21.53	66.96	27.37	63.23	18.71	66.96	27.37	69.03	29.08	76.52	16.23
	Boosting	65.05	8.39	63.54	12.70	77.56	16.08	68.08	8.07	77.56	16.08	54.83	18.28	72.71	11.90
LR forward admission (9)	SVM	80.00	11.78	86.53	14.52	75.04	17.60	78.73	12.41	75.04	17.60	86.25	14.14	84.77	11.12
	RF	79.52	10.71	78.70	13.93	83.35	16.80	79.46	11.48	83.35	16.80	78.19	14.55	89.03	8.36
	RL	72.57	7.17	73.94	16.87	78.77	17.34	73.43	6.83	78.77	17.34	69.03	23.28	77.61	10.41
	Boosting	73.10	6.96	68.73	11.78	84.64	13.12	74.96	8.60	84.64	13.12	62.60	12.19	79.44	9.68
LR backward admission (12)	SVM	90.33	7.44	100.00	0.00	81.65	13.39	89.34	8.41	81.65	13.39	100.00	0.00	93.61	9.23
	RF	81.62	12.95	78.61	16.28	86.77	10.79	82.06	12.98	86.77	10.79	75.78	18.55	88.34	11.45
	RL	64.14	8.52	67.72	14.77	62.90	17.59	62.51	8.93	62.90	17.59	68.34	16.73	72.01	10.49
	Boosting	68.29	12.62	68.78	15.72	77.80	18.18	70.75	10.46	77.80	18.18	59.42	27.02	76.99	12.71

Average performance metrics in % (accuracy, precision, etc.) for binary classification to predict CKD progression in patients with AKI secondary to COVID-19. SVM: support vector machine; RF: random forest; LR: logistic regression; ROC: receiver operating characteristic; SHAP: shapley additive explanations; PCA: principal component analysis; AUC: area under the curve.
